# Peer review practices in academic medicine: how the example of orthopaedic surgery may help shift the paradigm?

**DOI:** 10.1007/s00264-023-05729-6

**Published:** 2023-03-01

**Authors:** George D. Chloros, Christos I. Konstantinidis, Anastasia Vasilopoulou, Peter V. Giannoudis

**Affiliations:** 1 Academic Department of Trauma and Orthopaedic Surgery, School of Medicine, University of Leeds, Leeds General Infirmary, Clarendon Wing, Floor D, Great George Street, Leeds, LS1 3EX UK; 2 Orthopedic Surgery Working Group, Society for Junior Doctors, Athens, Greece; 3 Korgialeneio Mpenakeio Hellenic Red Cross Hospital, Athens, Greece; 4 NIHR Leeds Biomedical Research Center, Chapel Allerton Hospital, Leeds, UK

**Keywords:** Academic medicine, Peer review practices, Manuscript blinding, Fairness, Orthopedic surgery

## Abstract

**Purpose:**

To establish the current peer-reviewed practices in the discipline of orthopaedic surgery and correlate these to the journal’s impact factor. Unfortunately, this is not receiving much attention and a critical literature gap in various disciplines; thus, determining the current practices in the discipline of orthopaedic surgery could provide valid insight that may be potentially applicable to other academic medicine disciplines as well.

**Methods:**

Orthopaedic surgery journals belonging to the Journal Citation Reports were queried, and the following was extracted: impact factor (IF) and blinding practices: single (SBPR), double (DBPR), triple (TBPR), quadruple (QBPR), and open (OPR) blinding review process and possibility of author-suggested reviewer (ASR) and non-preferred reviewer (NPR) options.

**Results:**

Of the 82 journals, four were excluded as they allowed submission by invitation only. In the remaining, blinding was as follows: SBPR nine (11.5%), DBPR 52 (66.7%), TBPR two (2.6%), QBPR zero (0%), and OPR three (3.8%), and in 12 (15.4%), this was unclear. ASR and NPR options were offered by 34 (43.6%) and 27 (34.6%) journals respectively, whereas ASR was mandatory in eight (10.2%). No correlation between IF and any other parameter was found.

**Conclusion:**

The rules of the “game” are unclear/not disclosed in a significant number of cases, and the SBPR system, along with the ASR (mandatory sometimes) and NPR, is still extensively used with questionable integrity and fairness. Several recommendations are provided to mitigate potentially compromising practices, along with future directions to address the scarcity of research in this critical aspect of science.

**Supplementary Information:**

The online version contains supplementary material available at 10.1007/s00264-023-05729-6.

## Introduction

The peer review system can be tracked back to 1731 in the Royal Society, with the first peer review model being used as early as in 1752 where a knowledgeable group of peers reviewed the manuscripts, and their recommendations would later influence the decision of the editor-in-chief [1]. It is considered to be the best available system and currently serves as the gold standard and gatekeeper of Science, despite several aspects of it being controversial [2].

Over the years, its process of blinding has evolved in different types which include single-blind peer review (SBPR), double-blind peer review (DBPR), triple-blind peer review (TBPR), quadruple-blind peer review (QBPR), and open peer review (OPR) (Table 1) [3, 4]. In general, it is perceived that DBPR and OPR offer higher objectivity and fairness and may increase review quality [5, 6]. However, each system has strengths and weaknesses [7], and which one is best still remains a debate.

**Table 1:** The different types of peer review. *SBPR*, single-blinded peer review; *DBPR*, double-blinded peer review; *TBPR*, triple-blinded peer review; *QBPR*, quadruple-blinded peer review; *OPR*, open peer review; *ID*, identity

Blinding/peer review type	Author ID known to reviewers	Reviewer ID known to author	Editor knows the ID of authors and reviewers	Authors and reviewers know the editor’s ID
SBPR	Yes	No	Yes	Yes
DBPR	No	No	Yes	Yes
TBPR	No	No	No	Yes
QBPR	No	No	No	No
OPR	Yes	Yes	Yes	Yes

Another controversial issue pertains to reviewer suggestion (author-suggested reviewers (ASR)) or exclusion (non-preferred reviewers (NPR)) option by the authors during the submission process. ASR may offer reduced cost and increased speed of the review but may increase bias or introduce fraud as there been several cases of abuse with authors impersonating peer reviewers and reviewing their own work, a phenomenon called “fake peer-review”(FPR) [8, 9]. Conversely, NPR may exclude some knowledgeable, fair but strict reviewers [10].

To the authors’ knowledge, there has been no study of the aforementioned controversial issues in orthopaedic journals, and there is very limited literature on the subject looking at specific disciplines [8]. This study has the following aims: (1) establish the current peer-reviewed practices in the discipline of orthopaedic surgery and correlate them to the journal’s impact factor; (2) given those findings, provide recommendations to improve the system in orthopaedic surgery; those findings and lessons learned may also be extrapolated to other academic medicine disciplines to address this critical gap in the literature.

It is hoped that this manuscript will help contribute to addressing some of the potentially correctable flaws of our currently imperfect peer review system which definitely have an impact in its integrity, fairness, and equality that are relevant to all parties involved (editors, authors, reviewers, and readers) in all fields of medicine.

## Materials and methods

The authoritative Journal Citation Reports™ (JCR) database of Clarivate Analytics was queried in March of 2022. The journal category of “orthopaedics” belonging to the science-citation index expanded was selected, and the name of the journal, its ranking, and its 2020 impact factor (IF) were extracted [11]. Furthermore, journals with submission by invitation only were excluded. For each journal, the official webpage or their partner’s webpage where the journal was hosted was thoroughly examined in order to extract information about the type of the peer review used for evaluating the submitted papers. For journals which did not have clear information or no information at all regarding their established peer review practices, the Journal was directly contacted via email prompting for a quick survey with questions regarding its peer review practices (Appendix 1—Online survey).

If no answer was received after seven days, a reminder email was sent and then at regular intervals. If no answer was received after four total attempts, the type of peer review for the journal was marked as “unclear.” All journals were categorized in one of the four peer review types presented in Table 1.

Furthermore, a sample email account was created and registered in the electronic submission system of each journal in order to access the submission process and investigate whether the journal offers/mandates do not offer the options of ASR or NPR. The maximum number of allowed ASR or NPR was also recorded, and if it was ten or more, it was recorded as “no limit.” The IF of the journals, obtained as described, was compared with the acquired data to investigate for potential correlations with the ASR or NPR options [11].

### Statistical analysis

A point-biserial correlation was made for the types of peer review of the journals in correlation with the journal’s IFs. Moreover, further analysis investigated potential correlations between the ASR and NPR practices and IF. *p* values below 0.05 were considered statistically significant. The software used for the data analysis was IBM Statistical Package for the Social Sciences (SPSS®) version 26.

## Results

### Orthopaedic journals

Out of the 82 orthopaedic journals from the JCR query, four were excluded since they allowed submission only with invitation (Clinics in Podiatric Medicine and Surgery, Foot and Ankle Clinics, Hand Clinics, and Orthopaedic Clinics of North America). The remaining 78 journals as categorized in the area of “orthopaedics” by the JCR were included for the data analysis and had a mean IF of 2.672 (range 0.500–7.000) (Table 2).

**Table 2:** The included journals based on the Journal Citation Reports™ (JCR) database of Clarivate Analytics®. *ISSN*, the International Standard Serial Number; *eISSN*, electronic ISSN; *ASR*, author-suggested reviewers; *NPR*, non-preferred reviewers; *JIF*, journal impact factor

Rank (by IF)	Full journal title	ISSN	elSSN	JIF	Peer review type	ASR practices	NPR practices
1	*Journal of Physiotherapy*	1836–9553	1836–9561	7.000	DBPR	Optional	Optional
2	*Osteoarthritis and Cartilage*	1063–4584	1522–9653	6.576	SBPR	Optional	Optional
3	*American Journal of Sports Medicine*	0363–5465	1552–3365	6.203	DBPR	Optional	Optional
4	*Bone & Joint Research*	2046–3758	2046–3758	5.853	DBPR	Optional	Optional
5	*Journal of Bone and Joint Surgery-American Volume*	0021–9355	1535–1386	5.284	DBPR	No option	No option
6	*Journal of Orthopaedic Translation*	2214-031X	2214-031X	5.191	DBPR	Optional	Optional
7	*Bone & Joint Journal*	2049–4394	2049–4394	5.082	Unclear	Optional	Optional
8	*Arthroscopy-The Journal of Arthroscopic and Related Surgery*	0749–8063	1526–3231	4.772	DBPR	No Option	No option
9	*Journal of Arthroplasty*	0883–5403	1532–8406	4.757	DBPR	No Option	No option
10	*Journal of Orthopaedic & Sports Physical Therapy*	0190–6011	1938–1344	4.751	DBPR	No Option	No option
11	*Cartilage*	1947–6035	1947–6043	4.634	DBPR	No Option	No option
12	*EFORT Open Reviews*	2396–7544	2058–5241	4.618	DBPR	Optional	Optional
13	*Knee Surgery Sports Traumatology Arthroscopy*	0942–2056	1433–7347	4.342	DBPR	Optional	Optional
14	*Clinical Orthopaedics and Related Research*	0009-921X	1528–1132	4.291	DBPR	No Option	Optional
15	*Spine Journal*	1529–9430	1878–1632	4.166	Unclear	No Option	No option
16	*European Cells & Materials*	1473–2262	1473–2262	3.942	OPR	Mandatory	Optional
17	*Acta Orthopaedica*	1745–3674	1745–3682	3.717	TBPR	Optional	No option
18	*Clinical Journal of Sport Medicine*	1050-642X	1536–3724	3.638	Unclear	No Option	No option
19	*Journal of Orthopaedic Research*	0736–0266	1554-527X	3.494	Unclear	Mandatory	Optional
20	*Spine*	0362–2436	1528–1159	3.468	TBPR	No Option	No option
21	*Connective Tissue Research*	0300–8207	1607–8438	3.417	SBPR	No Option	No option
22	*Brazilian Journal of Physical Therapy*	1413–3555	1809–9246	3.377	DBPR	Optional	Optional
23	*European Spine Journal*	0940–6719	1432–0932	3.134	DBPR	No Option	No option
24	*International Orthopaedics*	0341–2695	1432–5195	3.075	DBPR	No Option	No option
25	*Archives of Orthopaedic and Trauma Surgery*	0936–8051	1434–3916	3.067	DBPR	No Option	No option
26	*Physical Therapy & Rehabilitation Journal*	0031–9023	1538–6724	3.021	DBPR	Optional	Optional
27	*Journal of Shoulder and Elbow Surgery*	1058–2746	1532–6500	3.019	DBPR	No Option	No option
28	*Journal of the American Academy of Orthopaedic Surgeons*	1067-151X	1940–5480	3.008	DBPR	Optional	No option
29	*Global Spine Journal*	2192–5682	2192–5690	2.915	DBPR	No Option	No option
30	*Journal of Orthopaedics and Traumatology*	1590–9921	1590–9999	2.907	DBPR	Optional	Optional
31	*Gait & Posture*	0966–6362	1879–2219	2.840	SBPR	Mandatory	No option
32	*Foot & Ankle International*	1071–1007	1944–7876	2.827	DBPR	No Option	No option
33	*Journal of Knee Surgery*	1538–8506	1938–2480	2.757	DBPR	Mandatory	Optional
34	*Orthopaedic Journal of Sports Medicine*	N/A	2325–9671	2.727	DBPR	Mandatory	Optional
35	*Foot and Ankle Surgery*	1268–7731	1460–9584	2.705	DBPR	Optional	No option
36	*Journal of Hand Surgery-European Volume*	1753–1934	2043–6289	2.688	DBPR	No Option	No option
37	*Archives of Osteoporosis*	1862–3522	1862–3514	2.617	Unclear	Mandatory	Optional
38	*Injury-International Journal of the Care of the Injured*	0020–1383	1879–0267	2.586	DBPR	Optional	No option
39	*Journal of Orthopaedic Trauma*	0890–5339	1531–2291	2.512	Unclear	Optional	Optional
40	*Journal of Orthopaedic Surgery and Research*	1749-799X	1749-799X	2.359	SBPR	No Option	Optional
41	*Bmc Musculoskeletal Disorders*	N/A	1471–2474	2.355	OPR	No Option	Optional
42	*Journal of Pediatric Orthopaedics*	0271–6798	1539–2570	2.324	DBPR	No Option	No option
43	*Journal of Foot and Ankle Research*	N/A	1757–1146	2.303	OPR	No Option	Optional
44	*Orthopaedics & Traumatology-Surgery & Research*	1877–0568	1877–0568	2.256	SBPR	No Option	No option
45	*Physician and Sportsmedicine*	0091–3847	2326–3660	2.241	DBPR	Mandatory	Optional
46	*Journal of Hand Surgery-American Volume*	0363–5023	1531–6564	2.230	DBPR	No Option	No option
47	*Skeletal Radiology*	0364–2348	1432–2161	2.199	DBPR	Optional	Optional
48	*Knee*	0968–0160	1873–5800	2.199	DBPR	No Option	No option
49	*Joint Diseases and Related Surgery*	2687–4784	2687–4792	2.181	DBPR	No Option	No option
50	*Hip International*	1120–7000	1724–6067	2.135	SBPR	No Option	No option
51	*Orthopaedic Surgery*	1757–7853	1757–7861	2.071	DBPR	Optional	Optional
52	*Clinical Biomechanics*	0268–0033	1879–1271	2.063	Unclear	Mandatory	No option
53	*Journal of Hand Therapy*	0894–1130	1545-004X	1.950	DBPR	Mandatory	No option
54	*Prosthetics and Orthotics International*	0309–3646	1746–1553	1.895	DBPR	Optional	Optional
55	*Clinical Spine Surgery*	2380–0186	2380–0186	1.876	Unclear	No Option	No option
56	*Journal of Hip Preservation Surgery*	2054–8397	2054–8397	1.872	DBPR	No Option	No option
57	*Geriatric Orthopaedic Surgery & Rehabilitation*	2151–4585	2151–4593	1.870	DBPR	No Option	No option
58	*Journal of Orthopaedic Science*	0949–2658	1436–2023	1.601	DBPR	No Option	No option
59	*Journal of Children’s Orthopaedics*	1863–2521	1863–2548	1.548	DBPR	No Option	No option
60	*Acta Orthopaedica et Traumatologica Turcica*	1017-995X	1017-995X	1.511	DBPR	No Option	No option
61	*Journal of Plastic Surgery and Hand Surgery*	2000-656X	2000–6764	1.462	DBPR	No Option	No option
62	*Journal of Back and Musculoskeletal Rehabilitation*	1053–8127	1878–6324	1.398	SBPR	Optional	No option
63	*Orthopedics*	0147–7447	1938–2367	1.390	DBPR	No Option	No option
64	*Journal of Foot & Ankle Surgery*	1067–2516	1542–2224	1.286	DBPR	No Option	No option
65	*Indian Journal of Orthopaedics*	0019–5413	1998–3727	1.251	DBPR	No Option	No option
66	*Operative Orthopadie und Traumatologie*	0934–6694	1439–0981	1.154	SBPR	Optional	No option
67	*Journal of Orthopaedic Surgery*	1022–5536	2309–4990	1.118	DBPR	No Option	No option
68	*Orthopade*	0085–4530	1433–0431	1.096	DBPR	No Option	No option
69	*Sportverletzung-Sportschaden*	0932–0555	1439–1236	1.077	Unclear	No Option	No option
70	*Journal of Pediatric Orthopaedics-Part B*	1060-152X	1473–5865	1.041	Unclear	No Option	No option
71	*Hand Surgery & Rehabilitation*	2468–1229	2468–1210	0.969	DBPR	Optional	Optional
72	*Zeitschrift fur Orthopadie und Unfallchirurgie*	1864–6697	1864–6743	0.923	Unclear	No Option	No option
73	*Orthopaedic Nursing*	0744–6020	1542-538X	0.913	DBPR	No Option	No option
74	*Journal of the American Podiatric Medical Association*	8750–7315	1930–8264	0.675	DBPR	Optional	Optional
75	*Acta Chirurgiae Orthopaedicae et Traumatologiae Cechoslovaca*	0001–5415	N/A	0.531	Unclear	Unclear	Unclear
76	*Isokinetics and Exercise Science*	0959–3020	1878–5913	0.519	SBPR	Optional	No option
77	*Acta Ortopedica Brasileira*	1413–7852	1809–4406	0.513	DBPR	No Option	No option
78	*Acta Orthopaedica Belgica*	0001–6462	0001–6462	0.500	DBPR	Optional	No option

### Peer review type

**Fig. 1:**
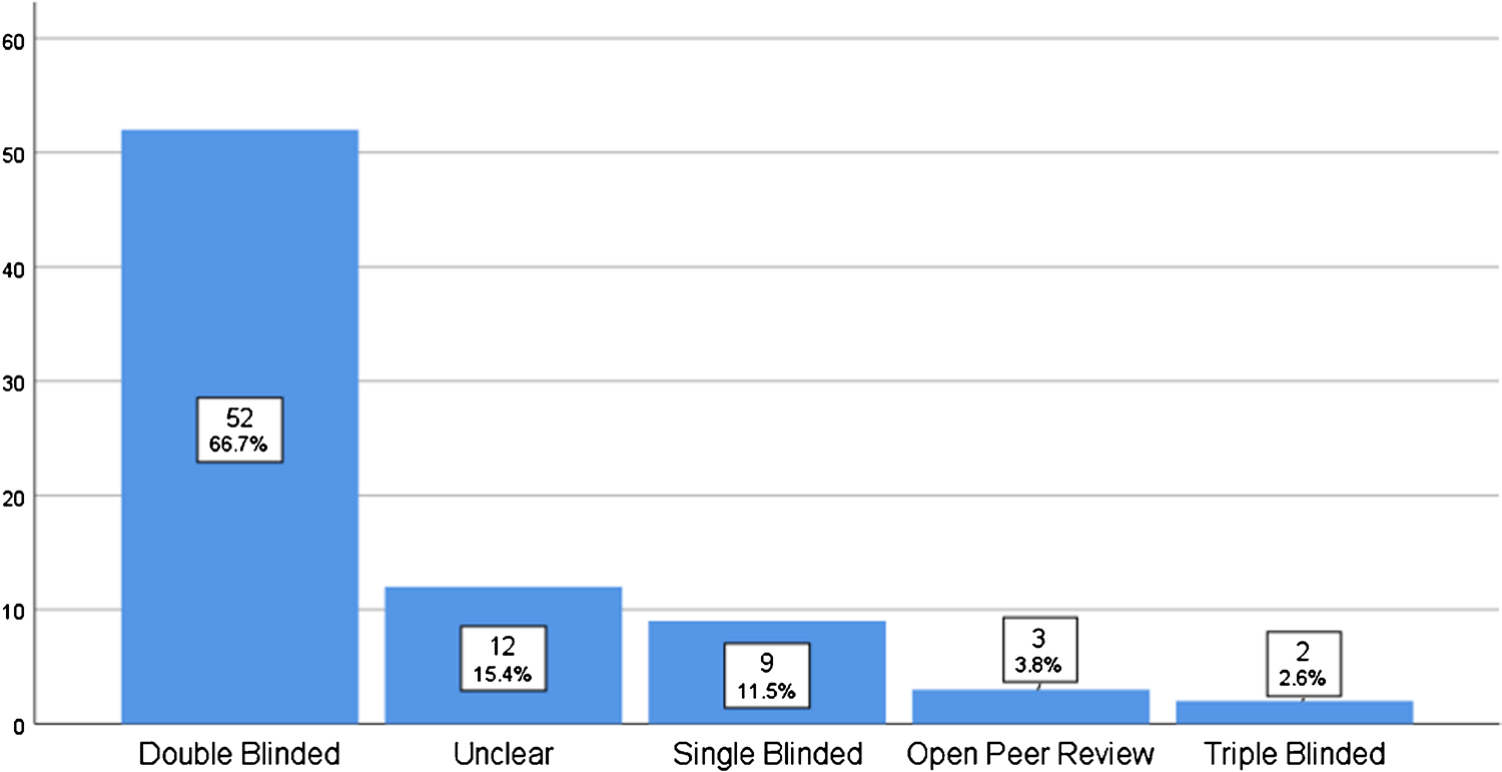
Peer review blinding practices of orthopedic journals (in decreasing order)

Only 47 of the 78 journals (60.3%) had a clear statement of their peer review type on the website. From the remaining 31 journals queried by email, 20 responses were received (64.5% response rate), with 19 accepted responses and one invalid response, with a total of 11 journals not responding at all after four attempts. Of the 78 journals, nine journals used SBPR (11.5%) (Table 2), 52 journals used DBPR (66.7%) (Table 2), two journals used TBPR (2.6%) (Table 2), three journals used OPR (3.8%) (Table 2), and in 12 journals, this was unclear (15.4%) (Table 2, Fig. 1). None of the journals used QBPR. A point-biserial correlation was made for the types of peer review vs IFs, but there was no statistically significant correlation between any type of peer review.

### Reviewer options (ASR and NPR)

In 77 of 78 journals, information regarding the optional choice or mandatory requirement of ASR or NPR during the submission process was acquired.

#### ASR

**Fig. 2:**
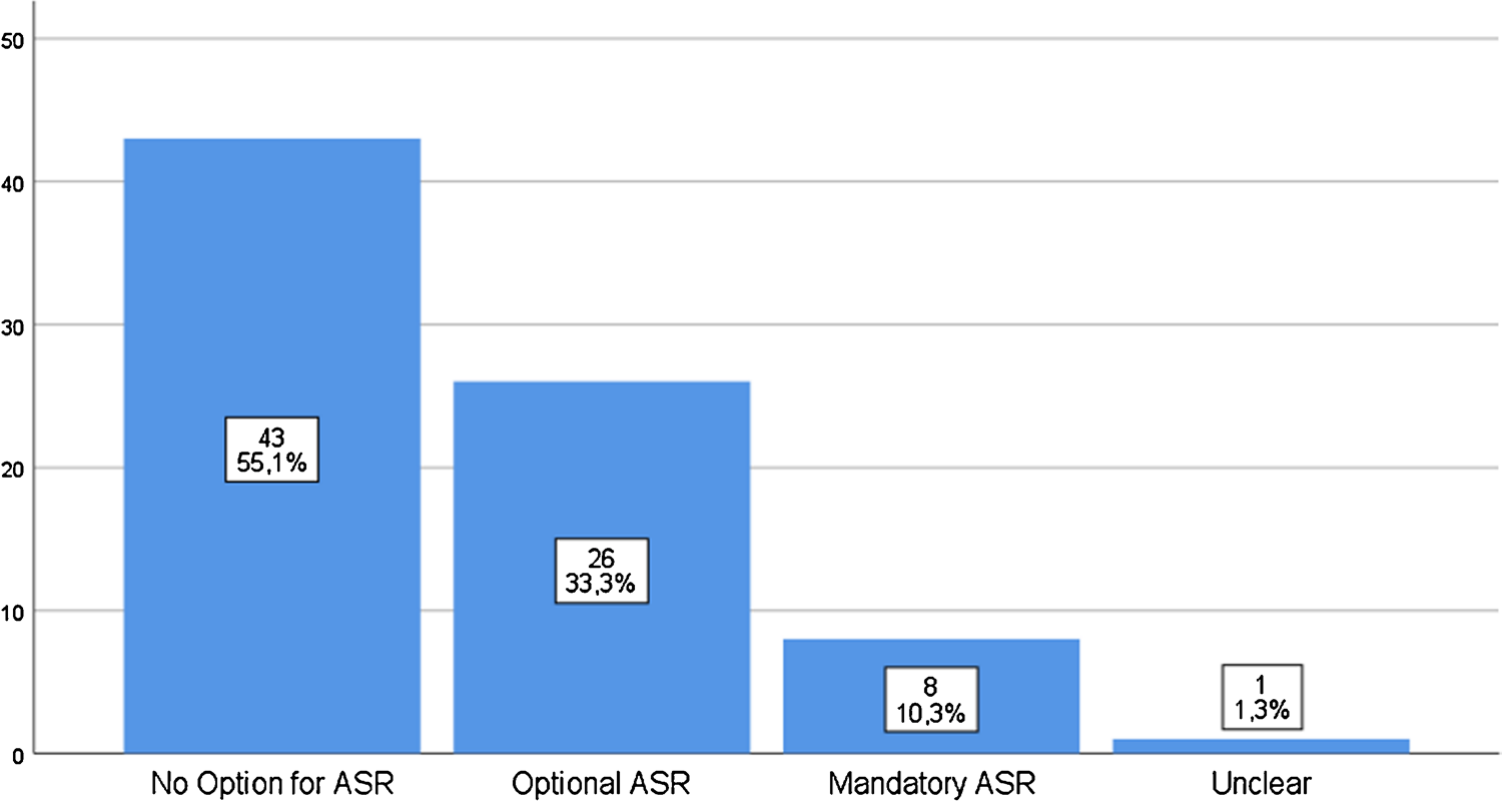
ASR practices by orthopaedic journals (in decreasing order)

Forty-three journals (55.1%) did not offer the ASR option, whereas 34 journals (43.6%) did, and for one journal, this was unclear. From the 34 journals that included the ASR, in 26, it was optional, whereas in eight, it was mandatory in order to proceed with the submission process (Table 2, Fig. 2). In 29 journals (85.3%), there was no limit in the number of ASR, and in four, it was limited to up to three reviewers (11.8%), whereas one journal allowed the suggestion of up to five reviewers (2.9%).

A point-biserial correlation was performed with coefficient of 0.205 (*p* = 0.071) for the option of ASR vs IF, but no statistically significant correlation was shown. Of the eight journals with mandatory-ASR, five required suggestions of at least two reviewers (62.5%), two required at least three reviewers (25%), and one required at least one reviewer (12.5%). There was no correlation between IF and the maximum number of ASR as well.

#### NPR

**Fig. 3:**
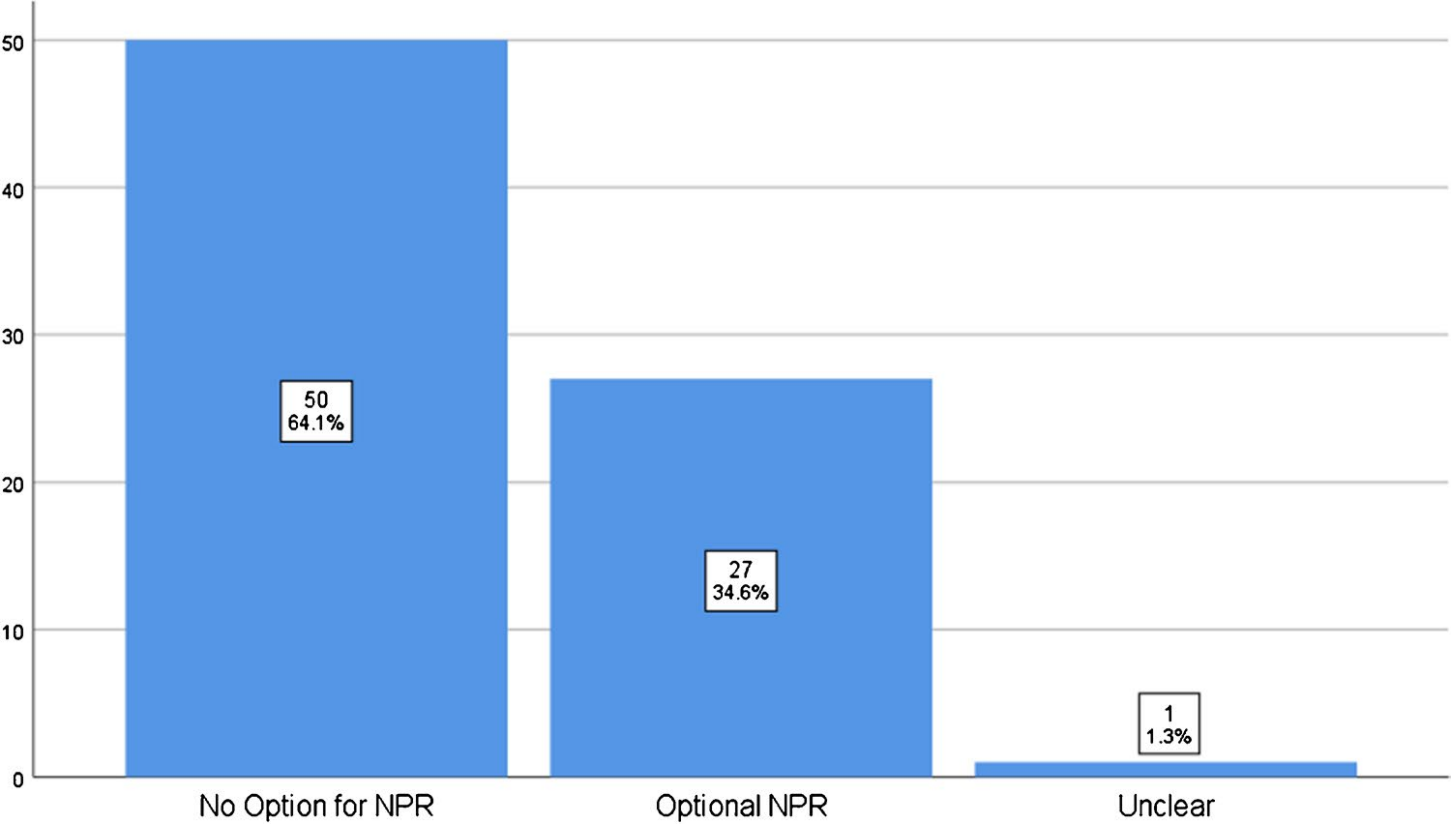
NPR practices by orthopaedic journals (in decreasing order)

Of the 77 journals only 27 (34.6%) allowed the author to be able to suggest NPR during the submission process (Table 2, Fig. 3). In 24 (88.9%), there was no limit in the number of NPR, in two, the limit was up to three reviewers (7.4%), and in one, the limit was up to five reviewers (3.7%). A point-biserial correlation coefficient of 0.331 (*p* = 0.003) indicated a statistically significant positive correlation about offering the option to suggest non-preferred reviewers and the journals’ IF. However, no significant association was observed on point-biserial correlation between number of allowed non-preferred reviewers vs journal IF.

## Discussion

Despite challenges, imperfections, and yet little proof that it works [12], the peer review system still remains the gold standard that safeguards the integrity of science [2]. Furthermore, although Dr. Lachmann quoted that “peer-review is in science what democracy is in politics, i.e. not the most efficient system, but the least corruptible” [13], there can still be unfairness, corruption, fraud, and lack of integrity. It is therefore important to have a clear picture of the peer-reviewed practices within a certain discipline for all parties involved and continuously strive to improve the ethical standards by stimulating objectivity and fairness. This study indicates that 2/3 (66%) of orthopaedic journals use the DBPR system, with the SBPR system following with 11.5%. By removing the 15.4% “unclear” component, DBPR rises up to 79%, SBPR to 13.5%, and the remaining OPR and TBPR with small percentages to 4.5% and 3% respectively. This “unclear” component is important, as potential authors and reviewers may not know if their information is shared during the peer review process. The authors of this paper recommend that all journals should have a clear indication of their peer review practices on their website and also in their electronic submission system, so that the rules of the game are disclosed to all parties, therefore ensuring better transparency.

In SBPR, the authors’ identities are revealed to the reviewers, and therefore, it has been criticized by many that it lacks fairness and impartiality. When authors that are previously well known and/or originate from prestigious institutions may receive favourable critiques, the inverse may be true for junior, less known investigators [3, 4, 14]. Additionally, further discrimination may be present secondary to ethnic origin, native language, institution, etc. [15]. We found that at least one in ten orthopaedic journals still employs SBPR, but no correlation with the IF was found. This is encouraging, as a recent study in the discipline of medical imaging showed that this was employed by 52% of the journals, and interestingly enough, journals with higher IF used this model more frequently [8]. Another study looking at dental journals showed that journals with a higher IF were most likely to have SBPR in place compared to the lower IF which was DBPR [16]. Given the obvious concerns raised above, the reasons for the persistence of SBPR are not clear. Interestingly, a 2016 study using a single orthopedic journal showed that when prestigious authors submit a manuscript, acceptance rate was 87% vs 68% in the case of SBPR vs DBPR, with higher reviewer ratings in all categories [14]. Based on the current study, the authors suggest that SBPR should be upgraded to DBPR as a first step to increase fairness of the submission process.

DBPR aims at eliminating the aforementioned biases, and this study found that it is the predominant system used by orthopaedic surgery journals (66.7%), which contrasts with some other medical disciplines [8]. Taking it to other fields, a survey of 590 chemistry journals showed that 97% did not embrace DBPR in 2007 [17], but this seems to be shifting just recently [18]. However, there are still limitations and remaining concerns with the DBPR: (1) Some studies (e.g., systematic reviews and randomized controlled trials) may have been registered in some public databases [19] or deposited in pre-print servers (e.g., medRxiv, www. https://www.medrxiv.org/) prior to peer review; (2) After removing the authors’ names and institutions, it is logistically sometimes difficult and time consuming to remove all blinding information; for example, it may be difficult to truly blind all manuscripts in a way that does not hint the reviewer, including removing discriminative self-revealing phrases such as “we have shown that” [4, 20] and also self-citations [21]. To theoretically mitigate those concerns, scrutiny by the editorial team of every manuscript demands resources, increases costs, may result in increased publication times, and is technically difficult [4]. Despite this fact, in a frequently cited survey by Ware and Monkman, 56% voted in favor of the DBPR versus 15% for the SBPR [22], and 80% of reviewers in nursing journals preferred DBPR with high levels of satisfaction [5]. Based on the above, and to ensure proper DBPR, as well as to lower administrative burden, journals should clearly state in the instructions to authors that sentences and/or hints that may potentially betray the authors’ identity should be excluded from the manuscript.

In DBPR, the editor still knows the authors’ identities, and therefore, this still carries a risk of bias. Therefore, theoretically, by automatically deidentifying this information during the submission process, additional blinding of the editor may be achieved through the so-called TBPR. The current study showed that this practice has only been implemented by two of the orthopaedic journals (Table 2) (2.6%). Possible reasons for this might be that, on one hand, this would require significant increase in the responsibilities and workload of administrative staff, and not all journals can handle that [3], and on the other hand, this might not be feasible when close communication between authors and editors is required [23]. Similar to the case of the DBPR, the authors speculate that the TBPR would require even more administrative resources. Furthermore, QBPR may be achieved by deidentifying the handling editor, to minimize the so-called “desk-rejections,” which take place as soon as the paper reaches one of the editors [24]. In this study, no orthopaedic journal has been found using this system.

In OPR, the identities of all the parties are known, and in some cases, all documentation of the entire peer review process, along with the names of reviewers, is fully available in the final published version of the manuscript [25]. Advocates of OPR often state that there is more accountability, and the reviews are increasingly constructive, have better quality, and promote a culture of “partnership” in publication [3]. Furthermore, OPR is a valuable resource for researchers who study the peer review system and also provides a better recognition of reviewer’s contribution to the manuscript [26]. However, there are concerns with OPR as far as a lower reviewer invitation acceptance rate [27] increased fear of retaliation especially for junior researchers and less critical feedback as reviewers may be reluctant to be strict [28]. Only three orthopaedic journals (3.8%) were found to practice OPR which shows a reluctancy to transition to this system (Table 2). Wolfram et al. in their 2018 study identified that there is a trend towards increasing the number of OPR journals since 2001 (174 vs 38 respectively), and it was mostly medicine and natural sciences that were adopting this model [29]. Of note, the “transparency” of OPR slightly differs between journals due to different OPR implementations. It is paramount thus that OPR journals clearly outline their open peer review process providing adequate information regarding the OPR implementation, the decision making process, and the editorial transparency [30].

There is currently a peer review crisis [2], in which 75% of journal editors reported that the hardest issue is finding reviewers to do the work [31], and that 4.70 invitations would be required for one to be accepted [32]. Therefore, the option of ASR to resolve those logistics would be compelling. However, this may come at a potentially enormous cost. Firstly, the mandatory option of ASR may raise some ethical questions as the authors are coerced to interfere with the peer review process and that would be a violation of their rights [9]. Second, the acceptance rate is higher, as authors may suggest friends and colleagues who would provide more favourable reviews, therefore compromising fairness [33, 34]. Last, but not least, FPR may account for at least 15% of retractions since 2012 [35], and only some few examples include Springer nature retraction of 107 articles from a single journal, 64 articles throughout ten journals [36], and SAGE retraction of 60 papers from a single journal [37]. Furthermore, Wang et al. reported that FPR was the most frequent cause for retraction in open access journals with the highest number of retractions and especially in journals with impact factor (IF) less than two [38]. The true magnitude of this problem cannot be calculated as it is impossible to detect all the cases, which poses a serious credibility issue to science [35]. Therefore, the editorial practice of asking (or even mandating) ASR during the submission process may compromise the journal, affecting its quality and, theoretically, its impact factor in the long term. This study found that 43.5% of the journals allowed ASR, and from these journals, 23.5% had this option as a mandatory requirement. We speculate that some of these journals may have a smaller pool of reviewers, and therefore, it is hard for them to find reviewers; however, this is an alarming finding, as the validity of the peer review system in orthopedic surgery may be thus compromised. To help eliminate bias, the authors recommend to eliminate the ASR option and, at a minimum, have the reviewers explicitly state their relationship(s) with the authors and vice versa (e.g., past mentors or past co-authors).

Noteworthy, this study also found that 34.6% of the journals allowed NPR options for the submitting authors. The option of having NPR correlated positively with the IF of the journals. Although it seems to have noble motives in eliminating reviewers with potential conflicts of interest, it can also be used unethically to increase the chances of publication, by deliberately rejecting specific knowledgeable reviewers that are known for their strict judgment and would have reviewed the paper in a fair but negative manner [10]. In turn, reviewers have an ethical obligation to alert the editor of their potential conflicts of interest and turn down the invitation to review [39]. Given the above findings, we recommend that the authors submitting a manuscript should justify in detail their NPR choice(s), and editors should only accept them based on solid grounds (even by querying the NPR in cases of doubt).

This study has some limitations: (1) The criterion for including an orthopaedic journal was its participation in the ISI Web of Science Journal Citation Reports which provides the journal information and official IF. The non-indexed journals were not evaluated. Nevertheless, this study gives a good idea of the baseline practices of those journals indexed in this prestigious database which could also be used to monitor any future changes. (2) Although correlations with the IF were made, and the journals were listed in decreasing IF ranking in Table 2, it is well known that the IF should not be looked at as an absolute measure of a journal’s quality [40]. (3) This study used information publicly available in the journals’ websites and sent survey-emails to the editors of journals with no online available information; no other means were considered to contact the non-responders (eg. telephone or social media). (4) This study answered quantitatively how many orthopaedic journals are using DBPR; however, it did not answer the question as to how effectively DBPR is achieved as alluded to previously. (5) This study did not compare orthopaedic surgery with other disciplines, because of scarcity in the literature. All three of these last limitations may be the objective of future studies.

## Conclusion

This study addresses serious issues in peer-reviewed practices in academic medicine that are not yet known for individual disciplines, let alone the fact that they have not yet been resolved. By investigating the paradigm of orthopaedic surgery, several alarming challenges are identified. Although currently two thirds of orthopaedic journals use DBPR, still one in ten uses less objective SBPR, and about four in ten allow ASR all of which may introduce bias and increase the potential for fraud. Unexpectedly, in 15% of cases, the model used is unclear, and the authors and reviewers may thus be unaware of the “rules of the game.” These findings may vary among disciplines, e.g., medical imaging [8]; however, there is significant gap in the literature. It is urgent to conduct similar research in the future in other disciplines to discover, compare and contrast their similarities and discrepancies, and apply the lessons learnt to help ensure maximal fairness and objectivity across the board in academic medicine.

## Supplementary Information

Below is the link to the electronic supplementary material.

Supplementary file1 (DOCX 13 KB)
