# Supplementary material for: Peer review practices in academic medicine: how the example of orthopaedic surgery may help shift the paradigm?
Source: Int Orthop. 2023 Mar 1;47(5):1137–45. doi: 10.1007/s00264-023-05729-6 (PMC10079738; doi:10.1007/s00264-023-05729-6)
Supplement: Supplementary file 1 — Supplementary file1 (DOCX 13 KB) [file 264_2023_5729_MOESM1_ESM.docx]

**ONLINE SURVEY – PEER-REVIEWED PRACTICES OF ORTHOPEDIC JOURNALS**

**1. Name of Journal:** ___________________________________________________

2. Are the **identities** of **authors** disclosed to the reviewers?

□No □Yes

3. Are the **identities** of **reviewers** disclosed to the authors?

□No □Yes

4. Are the **identities** of **authors** disclosed to the editor handling the manuscript?

□No □Yes

5. Is there an option for **authors** to **suggest** specific **reviewers**?

□No □Yes

6. If yes, then what is the **maximum number of suggested reviewers**? (set 0 if none)

7. Is there an option for **authors** to **exclude** specific **reviewers**?

□No □Yes

8. If yes, then what is the **maximum number of excluded reviewers**? (set 0 if none)

**Supplemental Digital Appendix 2**: Online Survey
